# Carriers of *LRRK2* pathogenic variants show a milder, anatomically distinct brain signature of Parkinson’s disease

**DOI:** 10.1038/s43856-025-01330-7

**Published:** 2026-01-03

**Authors:** Jakub Kopal, Andrew Vo, Qin Tao, Tanya Simuni, Lana M. Chahine, Danilo Bzdok, Alain Dagher

**Affiliations:** 1 Centre for Precision Psychiatry, Division of Mental Health and Addiction, Institute of Clinical Medicine, University of Oslo, Oslo, Norway; 2 Department of Biomedical Engineering, Faculty of Medicine, McGill University, Montreal, QC Canada; 3 The Neuro - Montreal Neurological Institute and Hospital, McGill University, Montreal, QC Canada; 4 Department of Neurology, Northwestern University Feinberg School of Medicine, Chicago, IL USA; 5 Department of Neurology, University of Pittsburgh, Pittsburgh, PA USA; 6 Mila - Quebec Artificial Intelligence Institute, Montréal, QC Canada; 7 Department of Neurology and neurosurgery, McGill University, Montréal, QC Canada

**Keywords:** Parkinson's disease, Parkinson's disease, Rare variants

## Abstract

**Background:**

Pathogenic *LRRK2* gene variants are a major genetic risk factor for both familial and sporadic Parkinson’s dissease (PD), opening an unattended window into disease mechanisms and potential therapies. Investigating the influence of pathogenic variants in *LRRK2* gene on brain structure is a crucial step toward enabling early diagnosis and personalized treatment. Yet, despite its significance, the ways in which *LRRK2* genotype affects brain structure remain largely unexplored. Work in this domain is plagued by small sample sizes and differences in cohort composition, which can obscure genuine distinctions among clinical subgroups.

**Methods:**

In this study, we overcome such important limitations by combining explicit modeling of population background variation and pattern matching. Specifically, we leverage a cohort of 603 participants (including 370 with a PD diagnosis) to examine MRI-detectable cortical atrophy patterns associated with the *LRRK2* pathogenic variants in people with PD and carriers without Parkinson’s symptoms.

**Results:**

LRRK2 PD patients exhibit milder cortical thinning compared to sporadic PD, with notable preservation in temporal and occipital regions, suggesting a distinct pattern of neurodegeneration. Non-manifesting LRRK2 carriers show no significant cortical atrophy, indicating no structural signs of subclinical PD. We further analyze the relationship between aggregated alpha-synuclein in cerebrospinal fluid and atrophy. We find that those with evidence of aggregated alpha-synuclein experienced pronounced neurodegeneration and increased cortical thinning, possibly defining another aggressive PD subtype.

**Conclusions:**

Our findings highlight genetic avenues for distinguishing PD subtypes, which could lead to more targeted treatment approaches and a more complete understanding of Parkinson’s disease progression.

## Plain language summary

Parkinson’s disease is a brain disorder that affects movement, thinking, and daily functioning. While most cases have no clear cause, some people carry rare changes in a gene called LRRK2. Another important factor is a brain protein called alpha-synuclein, which can build up in harmful ways. In this study, we used brain scans from a large international project to understand how these genetic and protein markers relate to brain changes. We found that people with LRRK2 mutations had less brain damage than those with typical Parkinson’s disease, even when they had similar symptoms. This suggests that some people may have natural protection in the brain. Our findings could help doctors better understand different forms of Parkinson’s and guide more personalized treatments in the future.

## Abstract (web summary)

Kopal et al. analyze MRI data from a Parkinson’s Disease cohort to assess how LRRK2 gene variants shape brain structure. They find that LRRK2-associated Parkinson’s Disease shows a milder and anatomically distinct pattern of neurodegeneration compared to non LRRK2-associated Parkinson’s Disease.

## Introduction

Monogenic genetic Parkinson’s Disease (PD) is rare, but studying disease-associated pathogenic variants has helped identify biological processes generally involved in sporadic PD. Mutations in the gene coding for Leucine-Rich Repeat Kinase 2 (*LRRK2*) are the commonest cause of familial PD^1,2^. The inheritance is autosomal dominant with age-dependent penetrance, from 28% at age 59 to 74% at 79 years^3^. *LRRK2* has emerged as a potential therapeutic target in both LRRK2-associated but also sporadic PD (sPD). Prevalence of *LRRK2* pathogenic variants among PD individuals is estimated to be 1–5% and is substantially higher in certain ethnic subgroups^4^. More importantly, although LRRK2 PD is relatively infrequent, there is post-mortem evidence of upregulation of *LRRK2* activity in the brains of people with sPD^5–7^. Because the disease-causing allele is a toxic gain of function mutation, *LRRK2* may be an attractive therapeutic target for inhibitory agents, which are already in clinical trials^1^. Indeed, growing evidence suggests that *LRRK2* kinase inhibitors may offer neuroprotective benefits in PD by restoring lysosomal function. Therefore, gaining a deeper understanding of neurodegeneration in individuals with *LRRK2*-associated PD may help distinguish *LRRK2*-specific mechanisms from broader PD pathology, offering critical insights into disease heterogeneity, progression, and potential compensatory pathways. Understanding these mechanisms could also aid in the development of precise imaging biomarkers for tracking *LRRK2*-targeted therapeutic responses and refining personalized treatment strategies for PD.

PD associated with *LRRK2* pathogenic variants has a clinical picture similar to sPD. Patients develop levodopa-responsive parkinsonism with a mean age of onset in the sixth decade^8^. They show evidence of dopamine neuron degeneration on single-photon emission computed tomography dopamine transporter imaging (DATscan), similar to sPD^9^. However, important differences have also been noted. LRRK2 PD appears to be milder with slower progression^1,10^. More importantly, only 40–75% of LRRK2 PD patients show characteristic Lewy pathology at autopsy^3^, suggesting that neurodegeneration in LRRK2 PD sometimes occurs in the absence of synucleinopathy. Of the roughly one-third of patients without synucleinopathy at postmortem, some demonstrate Alzheimer-like pathology, while others have dopamine neuron loss without evidence or protein aggregation^3,11^.

While it is not currently possible to quantitatively measure central nervous system Lewy pathology in living humans, amplification techniques similar to those used in prion diseases have been developed to detect aggregated alpha-synuclein (asyn) in cerebrospinal fluid (CSF)^12^. A positive CSF seed amplification assay (SAA) for asyn indicates Lewy pathology with high sensitivity and specificity, aside from amygdala predominant asyn distribution^13,14^. In LRRK2 PD, CSF asyn SAA is congruent with postmortem examinations of substantia nigra synucleinopathy^15^ and may also detect evidence of synucleinopathy in non-manifesting at-risk individuals^12^. Consistent with postmortem studies, CSF asyn SAA is positive in roughly 67% of LRRK2 PD individuals^12^. A negative CSF asyn SAA in LRRK2 PD appears to be associated with milder motor manifestations and slower progression^16^, positioning asyn SAA as an important disease biomarker.

Magnetic resonance imaging (MRI) serves as a crucial tool for differential diagnosis of PD and exploring its neuropathological mechanisms. The most useful MRI-derived indicators of neurodegeneration so far include cortical thickness (from T1-weighted MRI) and susceptibility-weighted or diffusion-weighted MRI of the substantia nigra. However, there have been few reports on the MRI-detectable characteristics of LRRK2 PD^9^. The studies published to date suffer from small sample sizes (typically below *N* = 20), where the evidence is further obscured by group differences in key sociodemographic and clinical factors. The Parkinson’s Progression Markers Initiative (PPMI) has collected MRI data on an exceptionally large cohort of sPD and LRRK2 PD participants with and without CSF asyn SAA positivity, as well as non-manifesting carriers (NMC)^17^. This extensive dataset opens a new window of opportunity to advance our understanding of PD through comprehensive MRI analysis. However, the PPMI cohort also faces limitations commonly seen in LRRK2 studies. Specifically, there are important demographic differences between sPD and LRRK2 PD with respect to disease duration, subject age, age of onset, and scanning site, making direct comparisons challenging. Matching on all these variables would once again yield small sample sizes, while using all the data leaves one vulnerable to important confounds. Here, we propose a principled hierarchical approach with a propensity score matching analytical protocol to overcome these limitations. As a result, this pattern-matching study details cortical atrophy distribution in LRRK2 PD and NMC, and evaluates the influence of CSF asyn SAA status. We show that (1) it is possible to account for unmatched datasets and known confounds, (2) cortical and subcortical atrophy in LRRK2 PD is milder than sPD, with a distinct spatial pattern, and (3) asyn SAA status correlates with greater degree of tissue loss.

## Methods

### Participant cohort

Brain-imaging and clinical measurements were acquired as part of the Parkinson Progression Marker Initiative (PPMI). PPMI is a global landmark study, launched in 2010, aimed at identifying biomarkers to improve the diagnosis and progression tracking of Parkinson’s disease^17^ (https://www.ppmi-info.org). Each participating PPMI site received approval from its local ethics committee, and written informed consent was obtained from all participants at enrollment. The overall PPMI protocol was approved by a central Institutional Review Board at the coordinating center. All data used in this study were accessed in fully anonymized, de-identified form through the PPMI repository, including defaced MRI scans and no site-identifying metadata. In accordance with our institutional guidelines, no additional IRB approval or individual consent was required for this secondary analysis.

From that resource, we have considered four subgroups with available MRI in our cross-sectional analysis: sporadic PD (sPD), PD patients with *LRRK2* pathogenic variants (LRRK2 PD), LRRK2 non-manifesting carriers (LRRK2 NMC), and healthy controls (HC). Details on the genotyping methods used in PPMI are described elsewhere^18^. The sPD subgroup consisted of 293 PD patients without a known pathogenic mutation associated with PD (e.g., *LRRK2*, *GBA1*, *SNCA*, *Parkin*, *PINK1*). These patients were recruited to the study within 2 years of diagnosis and were not treated with dopaminergic medications during the first 6 months of enrolment. A subset of sPD patients participated in follow-up visits spanning 4 years, and their most recent imaging data were utilized for the analysis. The LRRK2 PD subgroup enrolled 77 *LRRK2* pathogenic variants carriers (age 65.0 ± 8.7 y.o., 44 males, 33 females) within 7 years of PD diagnosis and irrespective of dopaminergic treatment status. Patients with mutations in both *LRRK2* and *GBA1* genes were excluded. The LRRK2 NMC subgroup consisted of 94 *LRRK2* pathogenic variants carriers without a PD diagnosis (age 62.0 ± 6.9 y.o., 40 males, 54 females). Finally, 139 healthy controls (age 61.1 ± 11.7 y.o., 92 males, 47 females) were absent of both pathogenic mutations and neurological diseases.

All participants with a diagnosis of PD had evidence of abnormal dopamine transporter imaging on single-photon emission computed tomography imaging^16,17^. The LRRK2 NMC were specifically recruited as first-degree relatives of PD patients of Ashkenazi Jewish origin from participating PPMI centres^19^. Most common pathogenic variant of *LRRK2 was* G2019S followed by R1441G^16^.

All demographic information can be found in Tables 1 and 2.

**Table 1:** Dataset demographics, diagnosis, and LRRK2

	PD diagnosis	*LRRK2* variants	count	age	N° males	disease duration
sPD	yes	no	293	63.3 ± 9.5	181	2.2 ± 1.9
LRRK2 PD	yes	yes	77	65.0 ± 8.7	44	3.2 ± 2.2
HC	no	no	139	61.1 ± 11.7	92	x
LRRK2 NMC	no	yes	94	62.0 ± 6.9	40	x

Key participant characteristics are provided with respect to the diagnosis of Parkinson’s disease and the presence of *LRRK2* pathogenic variants. Age and disease duration are presented as mean ± standard deviation.

*sPD* sporadic Parkinson’s disease, *HC* healthy control, *NCM* non-manifesting carrier.

**Table 2:** Dataset demographics and SAA status

	% PD diagnosis	% *LRRK2* variants	count	Age	N° males	disease duration
asyn SAA positive	98	15	301	63.1 ± 9.3	189	2.3 ± 2.0
asyn SAA negative	25	14	155	62.5 ± 11.7	92	2.2 ± 1.9

Key participant characteristics are provided with respect to the asyn SAA status. Age and disease duration are presented as mean ± standard deviation. % PD diagnosis column corresponds to the percentage of asyn SAA positive participants that carry PD diagnosis.

### Image acquisition and processing

All participants underwent T1-weighted MRI that followed standardized procedures and acquisition parameters (https://www.ppmi-info.org/study-design/research-documents-and-sops/). Surface-based cortical and volumetric subcortical processing was performed with FreeSurfer version 7.2^20^ to estimate vertex-wise maps of cortical thickness and subcortical volume. These two measures, as opposed to cortical surface area, have been shown to be most sensitive to neurodegeneration in PD^21^. Resulting brain maps were visually inspected for quality control and then parcellated into 68 cortical parcels according to the Desikan-Killiany atlas^21^ and 14 subcortical parcels according to Harvard-Oxford atlas^22^. These region of interest measures were computed by averaging the values of vertices within each parcel. Given that subcortical volumes scale with head size, models that examined these measures additionally included total intracranial volume as a covariate^23^.

### Alpha-synuclein seed amplification assay

Alpha-synuclein seed amplification assay (asyn SAA) was used to detect the presence of pathogenic asyn aggregates in the cerebrospinal fluid (CSF) at the first visit of each participant. Details on the asyn SAA methodology and protocol used in PPMI have been previously described^12,24^. Each participant’s CSF sample was tested three separate times. A positive or negative asyn SAA was determined based on consensus, or lack thereof, across the three replicates. As a result, the asyn SAA provides a unique opportunity in our study to link asyn aggregation to the structural brain changes captured through MRI.

### Continuous subgroup matching via propensity score calculation

To prepare the ground for dissecting the complexities of the dataset in terms of population strata, we isolated comparable groups of healthy controls (i.e., without PD diagnosis and *LRRK2* pathogenic variants) and sporadic PD patients (e.g., with PD diagnosis and without *LRRK2* pathogenic variants). One of the most commonly used techniques for comparing participant groups is manually aligning participants based on age and sex. Balancing out subject groups, based on their population background variation, ensures comparability between the groups under study and reduces potential confounding^25,26^. However, this manual approach becomes increasingly impractical with every additional covariate of undesired variation that needs to be considered. Therefore, we implemented a more flexible propensity score framework that natively aggregates a potentially large number of heterogeneous person characteristics into a single balancing index^27^.

Propensity scores were calculated using a logistic regression model, where the disease status was regressed on sex (encoded as a binary variable: female = 0, male = 1), age (in years), site (encoded as dummy variables for each site location), and, where applicable, disease duration (in years). The logistic regression model yielded a propensity score for each participant, representing the probability of carrying the diagnosis given the covariates:

$${{\mathrm{PS}}}=P(y=1\left|C\right.)$$

where y is the PD diagnosis, while *C* denotes a collection of covariates (e.g., sex, age, site, and, where applicable, disease duration) to be accounted for.

Importantly, these propensity scores were based solely on the participant characteristics, that is, without access to input variables of primary scientific interest (i.e., brain region morphometry). Essentially, the propensity score distills each individual’s diversity characteristics into one number, facilitating more accurate and balanced group comparisons.

### Hungarian algorithm to align the propensity indexed participants into subgroups

After obtaining propensity scores for each HC and sPD participant through a logistic regression model, we proceeded to pair participants between the two groups under study using the Hungarian algorithm^28^. This optimization technique is designed to maximize the total similarity of pairing participants between the two groups (HC and sPD). We first constructed a cost matrix, where each entry represented the difference in propensity scores between a given sPD participant and a HC participant at hand. The Hungarian algorithm was then applied to this matrix to identify the optimal one-to-one matching, ensuring that the sum of differences in propensity scores across all matched pairs was minimized. Notably, in this matching process, we incorporated a caliper parameter to ensure that participants were only matched if their propensity scores were sufficiently close^27^. This approach modifies the cost matrix by assigning a prohibitively high cost to pairs exceeding the caliper checkpoint, ensuring that only those participants within the acceptable range are matched by pairing, thus improving the comparability and accuracy of the resulting samples (more details in Supplementary Fig. 6). As an important consequence, since not all participants had sufficiently close propensity scores to be matchable, not all participants were selected for the following analysis (cf. corresponding stacked bar plots in figures). Instead, we have selected only closely matching pairs of participants, ensuring that the differences in propensity scores were within the acceptable range defined by the caliper. By aligning participants according to their propensity scores, the matching process created two subgroup samples (sPD and HC) that were balanced with respect to background variation in the population. In other words, the selected pairs of sPD and HC participants displayed similarly distributed covariates, reducing potential confounding effects and improving the comparability of the groups for subsequent analysis.

### Linear model to quantify structural changes in PD

After careful selection of matching pairs of HC and sPD subjects, we specified and estimated a series of linear regression models to quantify brain changes occurring in PD. Specifically, we estimated cortical thickness for cortical regions and volume for subcortical regions using PD diagnosis as model outcome (encoded as a binary variable: without diagnosis = 0, PD diagnosis = 1), sex (encoded as a binary variable: male = 0, female = 1), age (in years), and site (encoded as indicator variables, one for each site location). Models directed at subcortical volume used total intracranial volume as an additional input covariate following previous research^23^. The linear regression model follows a specification that can be formally expressed as:

$${Y}_{i}={\beta }_{1}\times {\mathrm{PD}}\,{{\mathrm{diagnosis}}}_{i}+{\beta }_{2}\times {{\mathrm{age}}}_{i}+{\beta }_{3}\times {{\mathrm{sex}}}_{{{\rm{i}}}}+{\beta }_{4}\times {{\mathrm{site}}}_{{{\rm{i}}}}+({\beta }_{5}\times {{\mathrm{TIV}}}_{i})$$

where ${Y}_{i}$ corresponds to the regional morphometry of participant $i$ and ${\mathrm{TIV}}$ is total intracranial volume. The analysis focused on the beta coefficient for PD diagnosis, with other covariates included to adjust for external sources of variation and ensure an accurate estimation of the effect of PD diagnosis on cortical thickness or subcortical volume.

Overall, we estimated 68 separate linear models corresponding to 68 cortical regions defined by the Desikan-Killiany cortical parcellation^21^ and 14 separate linear models corresponding to 14 regions defined by Harvard-Oxford subcortical atlas^22^. These 82 total models allowed us to assess the observed structural brain changes associated with PD.

### Transferring the derived PD brain model to unseen subgroups

To further explore the relationships between brain morphometry, PD, and the *LRRK2* pathogenic variants, we utilized 82 linear models originally dedicated to the comparison between sPD and HC. These models characterize a general PD-related atrophy pattern grounded in a relatively well-powered  subgroup comparison. Rather than fitting new models within each genetically or biomarker-defined subgroup, we evaluated the expression of this PD-derived pattern in LRRK2 PD and other subgroups. This transfer-like analytical framework enabled us to assess how closely each subgroup conformed to the prototypical atrophy pattern of PD. Importantly, this approach assumes that neurodegeneration in sPD captures a biologically meaningful axis of PD-related brain changes that can serve as a reference yardstick across PD subtypes. This strategy improves interpretability and statistical power in smaller subgroups where re-estimating independent models would be underpowered and susceptible to instability. Specifically, we focused on three group comparisons: LRRK2 PD versus sPD, LRRK2 NMC versus HC, and asyn SAA positive versus SAA negative participants. In short, our approach makes the quantitative analysis of these under-sampled subgroups possible in the first place.

As described (cf. above), the first step in these more nuanced subgroups involved creating matched groups of participant subsets based on a set of population background characteristics. Hence, we again employed propensity score matching to balance the groups in a way that reduces the effect of relevant sources of background variation in the population. For the comparison of LRRK2 PD and sPD participants, variant status was regressed on sex, age, disease duration, and site. The same set of covariates, with asyn SAA status as the outcome, was used to compare asyn SAA positive and negative participants. Finally, variant status was regressed on sex, age, and site in the HC versus LRRK2 NMC comparison. Using the Hungarian algorithm to create pairs of participants matched on propensity scores minimized potential confounding and thus allowed for cleaner comparisons of brain morphometry across the different subgroups.

In the second step, we leveraged the robustness of the models trained on the larger sPD vs. HC comparison to generate reliable morphometric estimates across different subgroups. Carrying over the models trained in earlier steps, in the new subgroups, avoided the necessity of estimating new model parameters and thus addressed the low statistical power due to the small sample size. Consequently, we derived regional morphometry estimates for each matched participant in every participant from each subgroup.

### Testing morphometric predictions in data-poor subgroups at the whole-brain level

Estimating regional morphometry allowed us to compare these estimates against actual measures of regional morphometry. In other words, we assessed the discrepancies between predicted and observed brain structure changes within each subgroup. Specifically, in each subgroup, we calculated the difference between predicted and measured morphometry for each participant. We then averaged these differences across participants to obtain a single aggregate estimate of predicted tissue differences for each brain region in each subgroup. Finally, we used paired t-tests to determine whether the differences between predicted and observed morphometry were statistically significant across regions, providing insight into the extent of brain structure changes associated with each subgroup at the whole brain level.

We also used the regional distribution of these prediction differences to evaluate whether the observed deviations from expected morphometry aligned with or diverged from the typical spatial pattern of PD-related atrophy.

### Linking PD severity, cognition, and genetic risk to brain changes

To corroborate and further attach meaning to the brain morphometry estimates, we analyzed their association with several key characteristics routinely assessed in PD patients. Namely, we used the MDS-UPDRS part III, which is a comprehensive tool used to measure the severity of Parkinson’s disease. Here employed Part III of the UPDRS, which assesses motor manifestations, such as tremor, rigidity, and bradykinesia^29^. Furthermore, we also characterized every PD patient using the Montreal Cognitive Assessment (MoCA) screening tool, designed to assess cognitive function and detect mild cognitive impairment. It evaluates various cognitive domains, including memory, attention, language, and executive functions^30^. Finally, we also used polygenic risk scores (PRS) for Parkinson’s disease computed by the PPMI consortium for every patient. PRS quantify an individual’s genetic predisposition to the disease by aggregating the effects of multiple genetic variants associated with increased risk. The PD PRS has been shown to be increased for LRRK2 PD^18^, suggesting that it may amplify the disease process in *LRRK2* pathogenic variant carriers. The PD PRS was computed and provided by the PPMI consortium from variants previously identified through genome-wide association studies^31^. We here used the modified PRS with LRRK2 locus excluded.

We collected UPDRS III and MoCA scores for every selected subject from the sPD versus LRRK2 PD comparison. We then used Pearson’s correlation to quantify the linear association strength between these motor and cognitive test scores and region-wise estimates of brain morphometry across participants. To complement this detailed examination and provide more general description, we also correlated UPDRS III and MoCA scores with morphometry estimates averaged across all regions. We employed the same strategy with PD PRS, but this time we used partial correlation analysis, controlling for the first 10 genetic principal components (PCs) to account for population structure and minimize potential confounding effects.

### Statistics and reproducibility

Statistical analyses were performed using Python (v3.10) and the statsmodels (v0.14) and scikit-learn (v1.3.1) packages. Sample sizes for all groups and subgroups are reported in Tables 1 and 2, and matched cohort sizes are detailed in Figs. 2–5. Each participant represented a single biological replicate. Reproducibility of participant selection was ensured using a consistent propensity score matching workflow with predefined calipers across all subgroup comparisons. All statistical tests were two-sided, and false-discovery rate correction was applied where noted. All code is publicly available to enable full reproducibility (see Code availability), and PPMI data are accessible to qualified investigators upon application.

### Reporting summary

Further information on research design is available in the Nature Portfolio Reporting Summary linked to this article.

## Results

### Differences in key clinical variables within Parkinson’s disease dataset

**Fig. 1:**
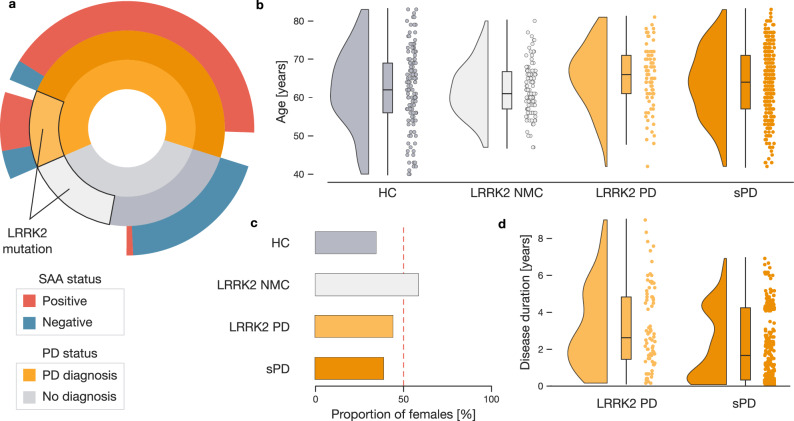
Complex interrelations between subgroups in ~ 600 participant population cohort. **a** Our participant sample consists of 370 participants with the diagnosis with PD diagnosis and 233 participants without PD diagnosis (inner circular bar plot). Among these, 94 non-PD participants and 77 PD patients carry *LRRK2* pathogenic variants indicated by the black edge (middle circular bar plot). Finally, the outer circular bar plot depicts the ratio of asyn SAA positive (red) and asyn SAA negative (blue) participants in each subgroup. **b** PD patients carrying *LRRK2* pathogenic variants represent the oldest subgroup. Raincloud plots display the age of each participant in one of the four subgroups. **c** Proportion of females among subgroups. Non-manifesting carriers with *LRRK2* pathogenic variants display the highest proportion of females. The red dotted line marks an equal ratio of male and female participants. **d** Different disease duration in PD patients. Disease duration is plotted for PD patients separated by the presence of *LRRK2* pathogenic variant. The diversity in key demographic metrics poses a challenge to comparing PD and control subgroups. Abbreviations: HC healthy controls, NMC non-manifesting carrier, PD Parkinson’s disease, sPD sporadic PD.

We set out to unravel the effects of *LRRK2* pathogenic variants as well as the effect of asyn SAA status in a generously profiled cohort of participants with PD and controls (Table 1). This cohort of 603 participants consisted of participants with sporadic PD and no known monogenic variant (*n* = 293), LRRK2 PD (*n* = 77), healthy controls (*n* = 139), and non-manifesting carriers of a *LRRK2* pathogenic variants (*n* = 94) (Fig. 1a). We further stratified our cohort based on CSF asyn SAA status. Across all participants, 296 displayed positive asyn SAA results, and 155 were asyn SAA negative (Table 2). The asyn SAA status was not available for the remaining participants.

The hierarchical constellation of participant attributes, along with the existence of overlapping subgroups defined by disease, variant, and asyn SAA status, posed an analytic challenge due to the differences in key demographic parameters among the subgroups. Specifically, for the subgroups defined by disease and variant status, the average age in the LRRK2 PD subgroup was 65.0 ± 8.7 years, while in LRRK2 NMC it was 62.0 ± 6.9 years (Fig. 1b). In addition, the LRRK2 NMC subgroup contained the highest proportion of females (57 %), while healthy controls (HC) contained the lowest proportion (34 %) (Fig. 1c). Finally, we noticed a difference in disease duration between sPD and LRRK2 PD; 2.2 ± 1.9 years vs. 3.2 ± 2.2 years (Fig. 1d). Besides the differences among the four subgroups defined by PD diagnosis and *LRRK2* status, further demographic differences existed among subgroups defined by asyn SAA status (Supplementary Fig. 1). Consequently, the complex relationship between key demographic factors called for a special approach to form homogenized and comparable subgroups for targeted analysis that is not unduly contaminated by unattended sources of general population variation^25,26^.

### Extracting a model of Parkinson’s disease brain atrophy

**Fig. 2:**
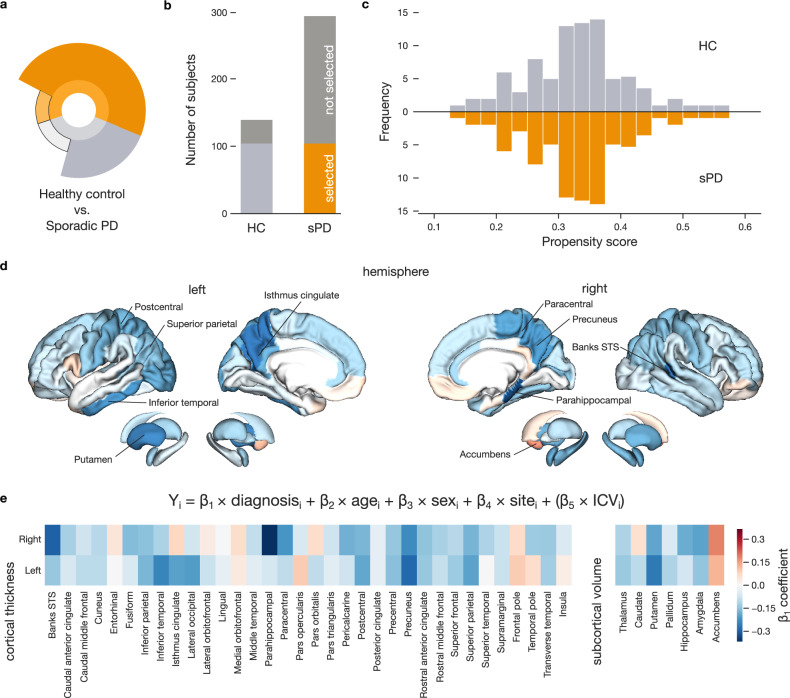
Region-specific models accurately estimate cortical thinning in PD. **a** The infographic shows the HC and sPD subgroups used to derive the reference model of PD-related brain alterations **b** Selection of carefully matched groups of participants. Only 104 out of 139 HC and 104 out of 293 sPD participants are carried forward to derive estimates of brain alterations associated with PD. **c** Propensity scores enable careful participant matching. Participants from both subgroups are matched based on age, sex, and site using a flexible propensity score matching strategy. The histograms display frequencies of propensity scores separately for sPD and HC subgroups. **d** Linear models incorporating diagnosis, age, sex, and site were used to predict cortical thickness and subcortical volume, with colored brain maps indicating regions where PD was associated with reduced (blue) or increased (red) structural measures. **e** Effect size of diagnosis across all brain regions. The heatmap shows diagnosis beta coefficients among 68 regions based on the Desikan-Killiany cortical parcellation and 14 regions defined by Harvard-Oxford subcortical atlas. Detected cortical thinning and volume loss aligns with established PD changes, underscoring our analytical protocol.

In order to overcome analytical challenges posed by the dataset structure, we first derived a quantitative model that explained PD as a function of structural brain alterations in carefully matched groups of sPD and HC participants (Fig. 2a). The matching strategy was based on propensity scores that considered age, sex, and site (cf. Methods). This approach guaranteed that PD diagnosis could not be predicted using these demographic factors alone. We thus selected 104 sPD and 104 HC matched participants for further analyses (Fig. 2b, c). For each brain region at hand, we estimated a separate linear regression model using PD diagnosis to predict regional thickness for cortical regions and regional volume for subcortical regions (Supplementary Fig. 2). Examining associated explained variance (R^2^, coefficient of determination) demonstrated that diagnosis accounted for a substantial portion of the variation in brain structure (Supplementary Fig. 3). The majority of the estimated linear regression slope coefficients yielded negative effects, indicating cortical and subcortical atrophy associated with PD diagnosis (Fig. 2d). We observed strongest atrophy effects in temporal and occipital brain regions, namely right parahippocampal gyrus (*ß* = −0.37), banks of the superior temporal sulcus (*ß* = −0.30), and left precuneus (*ß* = −0.29). Notably, the obtained general pattern of PD-related atrophy mirrored previous structural brain-imaging studies^23^. The effects of PD diagnosis were further exacerbated for participants with higher age (Supplementary Fig. 2). In summary, estimating a collection of dedicated linear models, applied to a carefully matched groups of sPD and HC participants, reliably captured expected, previously shown, structural alteration observed in PD patients.

### Delineating neurodegeneration changes associated with *LRRK2* pathogenic variants

**Fig. 3:**
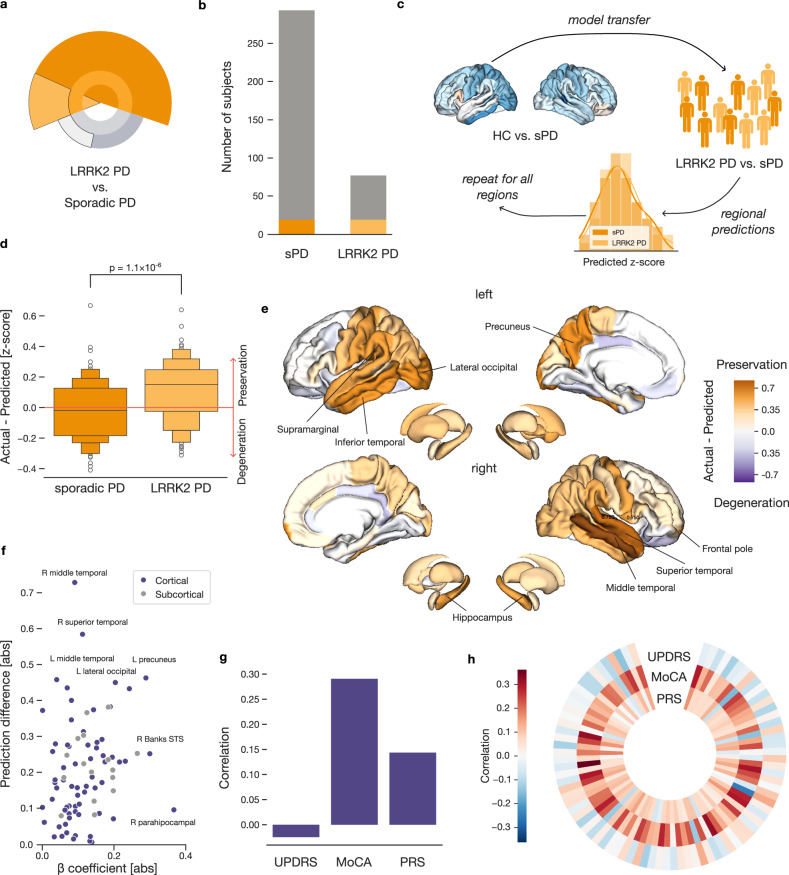
Parkinson’s patients with *LRRK2* pathogenic variants show a milder form of brain atrophy. **a** The infographic highlights the sPD and LRRK2 PD subgroups used to assess the extent of PD-related brain atrophy. **b** Selection of carefully matched groups of PD patients. Only 19 out of 293 sPD and 19 out of 77 LRRK2 participants were selected based on age, sex, disease duration, and site using propensity score matching. **c** Transferring the model of PD-related atrophy between cohort subgroups. We apply a PD atrophy model, trained on measures from sPD and HC subgroups, to estimate regional thickness and volume in LRRK2 PD and sPD patients. We then average these estimates to derive region-specific measures. **d** Model estimates reveal less atrophy in LRRK2 PD patients. The plot illustrates region-wise deviations between measured and estimated morphometry, with positive values indicating preserved structure and negative values reflecting greater degeneration. The p-value denotes a two-sample t-test of prediction differences. **e** *LRRK2* pathogenic variants are associated with less cortical thinning in temporal and occipital regions. Region-wise differences between measured and estimated brain metrics are plotted on cortical and subcortical regions for LRRK2 PD participants. Orange indicates less thinning or volume loss; purple denotes increased atrophy. **f** Relationship of prediction difference with PD atrophy. The diagnosis-related beta coefficients are plotted against prediction differences for every brain region. **g** Linking predictions of brain structure to cognitive and genetic measures. The correlations quantify linear association strength across all sPD and LRRK2 PD patients between averaged structural predictions and MDS-UPDRS III, MoCA, and PRS. **h** Relating regional predictions to cognitive and genetic factors. Correlations with regional predictions are shown for MDS-UPDRS III, MoCA, and PRS. Overall, patients carrying *LRRK2* pathogenic variants show less brain atrophy, with preservation especially in the temporal and occipital regions.

After estimating the PD-related atrophy in participants without *LRRK2* pathogenic variants, we directed our attention to the effects of these variants. To that end, we compared Parkinson’s patients carrying the *LRRK2* pathogenic variants against patients without this genetic status (Fig. 3a). We again carefully matched groups of sPD and LRRK2 PD participants using propensity scores, taking into account age, sex, disease duration, and site. We thus selected 19 patients with and 19 without the variants (Fig. 3b). To address the low statistical power due to the small sample size, we applied models trained in earlier steps using a larger participant set in these new subgroups, thus avoiding the necessity of estimating new model parameters. In other words, we carried the model of general PD atrophy over to these new subgroups defined by the presence of the *LRRK2* pathogenic variants. Specifically, we derived regional morphometry (i.e., cortical thickness and subcortical volume) estimates for each sPD and LRRK2 PD patient using the linear models derived in HC vs. sPD comparison. We then compared how these regional estimates differed from actual brain measurements (Fig. 3d). Finally, after averaging the differences between morphometry estimates and the actual measurements across all participants in each subgroup, we obtained a single measure of prediction difference for each brain region for the considered participants - sPD and LRRK2 PD (Supplementary Fig. 4). We observed a statistically significant difference between the two subgroups in these modeling outcomes (two-sided *t*-test *p* = 1.1 × 10^−6^). Notably in LRRK2 PD patients, the actual thickness and volumes were more preserved compared to those estimated by our model of PD atrophy pointing towards less cortical thinning and volume reduction in LRRK2 PD patients compared to sPD. As a sensitivity analysis, we repeated this comparison using only participants who were asyn SAA positive and observed nearly identical results, further supporting the robustness of our findings (Supplementary Fig. 5a). In conclusion, our participant matching strategy combined with the transfer of PD atrophy model revealed significantly milder brain atrophy in patients with *LRRK2* pathogenic variants.

To explore whether LRRK2 PD follows the same regional pattern of brain atrophy as sporadic PD, we investigated which regions showed the largest differences between predicted and measured morphometry. Positive differences, where measured morphometry exceeded model predictions, were interpreted as relative preservation of brain structure in LRRK2 PD. In general, regions with the highest preservation were located predominantly in the temporal and occipital lobe (Fig. 3e). Concretely, the largest preservations of thickness were observed in the left and right middle temporal gyrus, right superior temporal gyrus, or in the left precuneus.

To formally compare this pattern of preservation to the previously obtained pattern of PD-related brain atrophy (cf. above), we calculated Pearson’s correlation across the two sets of brain region measures (i.e., model coefficients and prediction differences mapped onto the brain). We used a spin-permutation test across the whole brain to examine the statistical significance of Pearson’s correlation between brain maps. The two brain maps were not statistically similar (*r* = 0.20, *p*_spin_ > 0.05), and especially regions such as right parahippocampal gyrus displayed large divergence between atrophy and preservation (Fig. 3f). In contrast, left precuneus and lateral occipital gyrus displayed substantial cortical thinning as well as preservation in LRRK2 PD patients. This suggests that the *LRRK2* pathogenic variants may be linked to a distinct spatial pattern of brain changes compared to typical sPD atrophy.

After establishing LRRK2 PD as a milder and distinct version of PD, we sought to explore how clinical severity of PD, individual differences in cognitive function, and genetic risk liability relate to brain structure predictions. Specifically, we examined how the Movement Disorders Society Unified Parkinson’s Disease Rating Scale (UPDRS) part III correlates with predicted measurements of expected brain morphometry to assess the impact of brain atrophy on motor manifestations. Furthermore, the relationship between the Montreal Cognitive Assessment (MoCA) scores and predicted morphometry provided insights into how global cognitive function in PD is linked to specific structural brain changes. Finally, associations with polygenic risk scores (PRS) for Parkinson’s disease allowed us to explore genetic risk to brain structure alterations in PD. In the first step, we used such predictions of brain thicknesses and volumes averaged across all regions for all LRRK2 PD and sPD patients (Fig. 3g). We observed significant Pearson’s correlation with MoCa, where decreased morphometry was linked to lower cognitive scores (*r*_MoCA_ = 0.29, *p*_FDR_ = 0.001). The other two tests did not reach significance (*r*_UPDRS_ = −0.03, *r*_PRS_ = 0.14 both *p*_FDR_ > 0.05). Collectively, this significant association links previously observed better cognitive performance of LRRK2 PD compared to sPD subjects and their reduced cortical thinning.

In the second step, we explored the relationship between MDS-UPDRS part III, MoCA, and PRS with predicted morphometry of each region separately. From a broader perspective, the associations followed the anticipated directionality (Fig. 3h). The strongest link with cognitive impairment represented by MoCA scores was with the postcentral gyri (*r*_left_ = −0.38; *r*_right_ = 0.26), the paracentral gyri (*r*_left_ = 0.33; *r*_right_ = 0.33), and the superior temporal gyrus (*r* = 0.32). Meanwhile, the left caudal middle frontal gyrus (*r* = −0.15) and the right putamen (*r* = −0.12) displayed the strongest link to motor manifestations (UPDRS) (Supplementary Table 2). Although the associations did not remain significant after FDR correction for the 82*3 tests, the uncovered patterns can guide future research efforts. In conclusion, these results demonstrated that LRRK2 PD patients exhibited considerably less cortical thinning and volume reduction compared to sPD patients, particularly in temporal and occipital regions. The observed association patterns between clinical severity, cognitive function, and brain structure offer valuable insights that may inform future research directions in understanding the nuances of Parkinson’s disease progression.

**Fig. 4:**
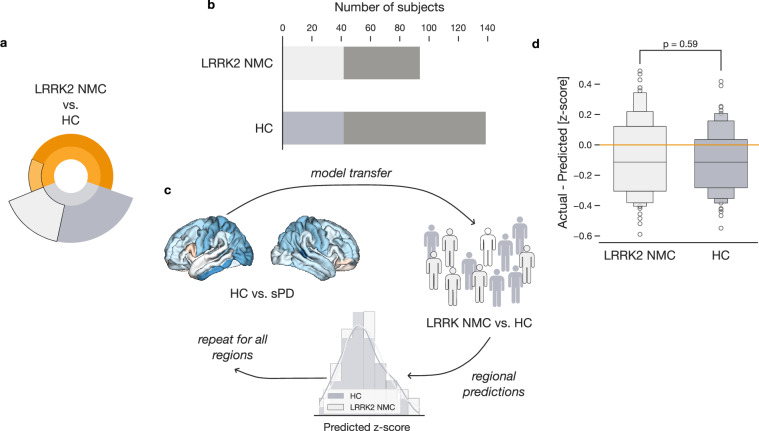
Absence of significant brain structure alterations in LRRK2 non-manifesting carriers. **a** We analyze the influence of *LRRK2* pathogenic variants in participants without PD diagnosis. **b** Selection of carefully matched groups of HC and LRRK2 NMC participants. Matched groups of 42 HC and 42 LRRK2 NMC were selected based on age, sex, and site. **c** Transferring the model of PD-related atrophy. We apply a PD atrophy model, trained on measures from sPD and HC subgroups, to estimate brain morphometry. **d** Comparable estimates of brain morphometry in controls. Boxen plots display differences between measured and predicted morphometry. The *p* value denotes a two-sample *t*-test of prediction differences. Unlike in PD patients, the presence of *LRRK2* pathogenic variants alone is not associated with significant structural brain alterations in participants without PD diagnosis.

In addition to studying the effects of the LRRK2 pathogenic variants in PD patients, we also assessed the impact of the variants on participants without a PD diagnosis. Analogous to previous steps, we first selected carefully matched subgroups of HC and LRRK2 NMC participants (Fig. 4a). Propensity score matching based on age, sex, and site indicated 42 closely matched pairs of participants without PD diagnosis (Fig. 4b). We again leveraged the opportunity to transfer readily-deployable models from the HC vs. sPD comparison (Fig. 4c). Therefore, we calculated regional morphometry for each HC and LRRK2 NMC participant using the pre-estimated models. We then compared the obtained regional estimates to actual brain measurements and averaged the differences across all participants in each subgroup (Supplementary Fig. 4). Based on the single region-specific estimates for HC and LRRK2 NMC subgroups, we did not observe a significant difference between these subgroups (two-sided *t*-test *p* = 0.59) (Fig. 4d). Our findings thus indicated that the *LRRK2* pathogenic variants do not profoundly affect brain structure in healthy participants without a PD diagnosis.

### Relationship between aggregated alpha-synuclein and brain atrophy in Parkinson’s disease

**Fig. 5:**
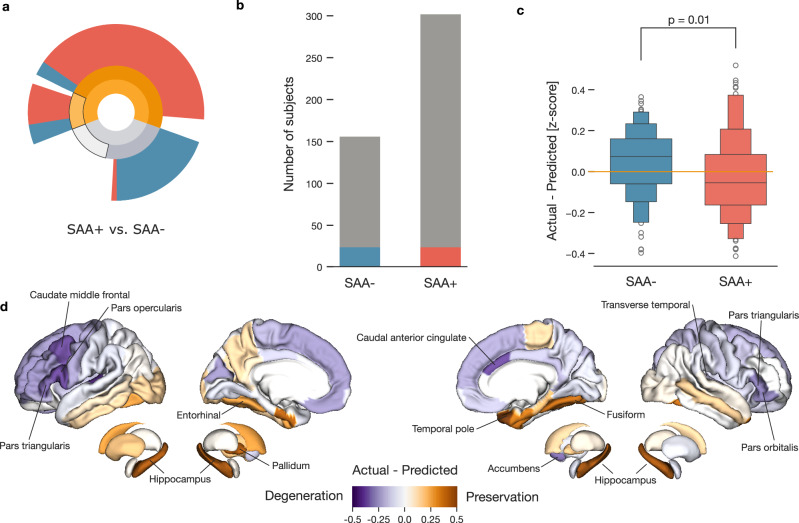
Aggregated alpha-synuclein proteins associated with increased brain atrophy. **a** We analyze the influence of asyn SAA measured in HC, sPD, and LRRK2 PD participants. **b** Matched groups of 23 asyn SAA negative and 23 asyn SAA positive participants were selected based on age, sex, disease duration, and site. **c** Greater atrophy in asyn SAA positive participants. Differences between measured and predicted morphometry reveal more pronounced degeneration in asyn SAA positive compared to negative individuals. **d** Differences between measured and predicted morphometry for asyn SAA positive participants are shown across the brain. Orange indicates regions with less atrophy than expected; purple shows greater thinning or volume loss. Our findings reveal pronounced cortical thinning specifically linked to asyn SAA positive status, with distinct cortical regions showing increased vulnerability.

Our final goal was to investigate the relationship between aggregated asyn and PD-related brain atrophy. Repeating previously utilized methodology, from all available participants, we selected 23 asyn SAA positive participants and 23 participants without that attribute, who we matched using propensity scores based on age, sex, site, and disease duration (Fig. 5a, b). Considering the small number of matching subjects, we transferred the model of PD-related atrophy to obtain an estimate of morphometry for each brain region in each matched participant (Supplementary Fig. 4). These estimates enabled us to examine how aggregated asyn in CSF relates to PD-related brain atrophy in rigorously matched participants.

Comparing obtained regional estimates to actual brain measurements revealed a significant difference between the asyn SAA positive and asyn SAA negative subgroups (two-sided *t*-test *p* = 0.01). Specifically, asyn SAA positive subjects displayed reduced thickness and lower subcortical volumes than predicted suggesting greater cortical thinning and volume reduction in the presence of the misfolded asyn (Fig. 5c). Notably, when comparing matched participants based on disease diagnosis and regardless asyn SAA status, we found no statistically significant differences (two-sided *t*-test, *p* > 0.05). This suggests that the observed morphometric differences between asyn SAA positive and SAA negative are not explained by diagnosis alone. Applying the same approach and stratifying by LRRK2 variant status yielded a borderline result (*p* = 0.05, uncorrected), suggesting that additional structural differences may exist between LRRK2 asyn SAA positive and negative individuals (Supplementary Fig. 5b, c). These potential differences warrant further investigation in larger, well-powered studies.

Further region-by-region inspection of the difference between estimated and measured structure in asyn SAA positive participants highlighted pronounced degeneration in the frontal and parietal regions (Fig. 5d). In particular, regions showing the greatest difference between estimated and measured structure included the left transverse temporal gyrus, left pars opercularis, right caudal anterior cingulate, and left caudal middle frontal gyrus. Conversely, a few regions, such as the right temporal pole and the subcortical regions, exhibited milder PD-related atrophy. Collectively, our results characterized the links of positive asyn SAA status with greater brain atrophy, which may reflect more severe underlying pathology and a poorer prognosis. These findings underscored the potential of CSF asyn SAA as a biomarker for monitoring or early detection of more severe neurodegeneration in Parkinson’s disease patients.

## Discussion

Understanding the impact of LRRK2 pathogenic variants that some individuals carry in their genome is critical, as they represent the most common genetic cause of PD and offer a unique window into its underlying biology^32^. In this pattern-matching study, we examined the impact of the *LRRK2* pathogenic variants on brain structure in individuals with and without a PD diagnosis. We found that LRRK2 PD patients exhibit milder cortical thinning and subcortical volume loss compared to matched sPD individuals. The preservation was particularly prominent in temporal and occipital regions. Among participants without PD diagnosis, LRRK2 NMC did not show significant structural differences relative to HC, suggesting no widespread subclinical atrophy. Finally, individuals with Lewy body pathology, inferred via asyn seeding assay, showed more pronounced cortical thinning, highlighting the influence of pathological subtypes on brain structure. These findings highlight avenues for distinguishing PD subtypes, which could lead to more targeted treatment approaches and a more complete understanding of PD progression.

Our analysis workflow began with the extraction of a core pattern of PD-related brain atrophy that captures comparisons between sPD and HC participants. The extracted brain pattern replicated previous findings of PD-related atrophy, particularly in posterior cortical regions^23^. Building on this validated pattern, we then used it as a benchmark to investigate structural changes in PD patients with the *LRRK2* pathogenic variants. Our findings revealed that LRRK2 PD patients exhibit milder cortical thinning and subcortical volume loss compared to sPD. Notably, this difference remained significant even when restricting the comparison to asyn SAA positive individuals, reinforcing that the observed structural preservation is attributable to the presence of *LRRK2* variants rather than differences in SAA status. Moreover, the pattern of cortical thinning and subcortical volume loss in LRRK2 PD differed from the previously identified PD-related brain atrophy and may represent a unique MRI signature. In particular, we observed reduced cortical thinning in posterior cortical areas and relative preservation of subcortical nuclei volumes. Recent evidence also documents a distinct brainstem MRI signature for LRRK2 PD subjects that differentiates them from both sPD and HC^33^. While neuronal loss in the substantia nigra pars compacta and locus coeruleus represents a hallmark of PD^34,35^, LRRK2 PD patients previously displayed preservation of locus coeruleus neuromelanin^33^. In addition, prior reports also documented significantly higher basal forebrain volumes for LRRK2 PD compared to sPD and as well as HC^36–38^. We here expand these findings with notable preservation of cortical tissue in the temporal and occipital lobes.

The preservation of cortical thickness and subcortical volume in LRRK2 PD subjects reported here also included nucleus accumbens and pallidum. Both regions are anatomically and functionally connected to the basal forebrain, a major source of cholinergic input to the cortex and subcortex. Previous studies have reported relative preservation of the basal forebrain in LRRK2 PD^36^. Taken together, these findings point toward a more intact cholinergic system in this subgroup. This view would align with the hypothesis that LRRK2-associated hypercholinergic activity may serve as a compensatory response to ongoing neurodegeneration^36^. Notably, elevated acetylcholinesterase activity has been observed in carriers of the *LRRK2* pathogenic variant even before symptom onset, suggesting a prodromal upregulation of cholinergic tone as a potential compensatory response to early neurodegeneration^37^. In addition to neuronal sources, acetylcholinesterase is also expressed by glial and immune cells^39^, indicating that cholinergic activity may interact with inflammatory processes in the brain. Importantly, cholinergic system changes in PD are not uniformly distributed but show regional heterogeneity, with certain hubs, such as the basal forebrain, hippocampus, and posterior cortical areas, playing disproportionate roles in compensatory responses^40^. This regional specificity may also help explain the relative preservation of posterior cortical areas in our LRRK2 PD cohort, as these regions receive cholinergic input from the basal forebrain and may benefit from compensatory signaling^41^. While these findings offer one plausible explanation, the biological mechanisms underlying the differential patterns of cortical atrophy in LRRK2 PD versus sPD remain incompletely understood. A more definitive understanding will require multimodal biomarker approaches, including proteomics, metabolomics, and other molecular profiling, many of which are already underway within the PPMI initiative. Clarifying these mechanisms will be critical for identifying compensatory pathways and advancing personalized intervention strategies tailored to individuals with preserved versus diminished cholinergic function.

Recently emerging evidence suggests that LRRK2 PD may represent a biologically distinct subtype of PD, characterized by both neuronal and clinical preservation. From a clinical standpoint, LRRK2 PD patients show milder cognitive symptoms in comparison to sPD^42^. We observed a strong positive relationship between the predicted brain-structure metrics and participants’ cognitive performance. This relationship aligns with earlier studies linking greater cortical thickness with higher MoCA scores^43^. Since the LRRK2 PD subjects displayed less cortical thinning in our sample, the structural preservation may help explain the known relatively better cognitive outcomes observed in this subgroup^44^. Given earlier findings of preserved cholinergic tone in LRRK2 PD, it is thus plausible that both structural and neurochemical resilience contribute to maintaining cognitive function in this subgroup^45^. Together, these findings would be in line with the idea that LRRK2 PD may represent a clinically relevant subgroup characterized by reduced loss of brain volume, which could inform stratified therapeutic approaches^19^.

We further examined whether *LRRK2* pathogenic variants are associated with subclinical neurodegeneration in individuals without a PD diagnosis. The analysis revealed that NMC did not exhibit significant cortical atrophy compared to healthy controls, suggesting an absence of a subclinical neurodegenerative process. This stands in contrast to individuals with prodromal PD, such as those with REM sleep behavior disorder, who frequently exhibit cortical atrophy well before the onset of motor or cognitive symptoms^46^. The absence of such atrophy in NMC is consistent with the reduced penetrance of *LRRK2* mutations and suggests that additional genetic, environmental, or biological factors may be required to trigger neurodegeneration in this group. Nevertheless, there is previous evidence of differences between HC and LRRK2 NMC, including increased volume in cuneus^47^ or smaller volume in the hippocampal region^48^ for LRRK2 NMC. Furthermore, LRRK2 NMC displayed significantly increased brain cholinergic activity measured by positron emission tomography^37^. We here also observed several notable differences between predicted and measured thickness in LRRK2 NMC, though these did not reach statistical significance. These observations thus call for more well-powered studies investigating whether asymptomatic subjects remain structurally unaffected despite the genetic risk. These studies will need to reexamine larger LRRK2 NMC cohorts based in SAA status and factoring degree of dopaminergic dysfunction.

Finally, to better understand the role of Lewy pathology in PD-related brain atrophy, we analyzed structural differences in participants stratified by asyn SAA status—a proxy for the presence of Lewy bodies. Our analysis revealed that asyn SAA positive participants exhibited greater cortical thinning than asyn SAA negative individuals. Similarly, a recent study documented that volumetric differences from healthy controls were less pronounced in PD-SAA negative, potentially indicating a milder neurodegenerative process^49^. These findings support the interpretation that asyn aggregation is associated with more aggressive neurodegeneration, potentially through mechanisms that directly damage cortical tissue. Specifically, the greater cortical thinning observed in asyn SAA positive individuals may reflect the neurotoxic effects of asyn accumulation, which has been linked to synaptic dysfunction, inflammatory cascades, and progressive neuronal loss in vulnerable cortical regions^50,51^. Notably, only two-thirds of LRRK2 PD patients show evidence of asyn aggregates compared to 90+% of sPD individuals^3,34^. Experimental studies indicate that LRRK2 overexpression can exacerbate asyn aggregation and toxicity, while *LRRK2* inhibition or knockout mitigates asyn-induced neurodegeneration^52^. However, the precise molecular mechanisms underlying the interaction between LRRK2 and asyn remain incompletely understood and warrant further investigation^53^. Similarly, because our asyn SAA positive and asyn SAA negative groups differed in age, sex, education, and disease duration, the propensity score algorithm could only match a small fraction of the participants. Therefore, larger-scale studies are needed to elucidate how asyn aggregation and *LRRK2* pathogenic variants interact to shape distinct patterns of neurodegeneration. In summary, these findings align with emerging evidence that PD may not correspond to a single, monolithic disorder but a family of neurodegenerative syndromes, unified by overlapping motor and non-motor features yet diverging in progression rates, affected brain regions, and underlying biochemical and molecular pathologies^54^.

Studying structural brain changes in LRRK2 PD and other rare genetic forms of PD (e.g., *PRKN*, *PINK1*, *GBA*, or *SNCA*) is challenged by the small sample sizes and heterogeneity of available cohorts in terms of participants' population background^9,55,56^. These limitations often restrict analyses to a few preselected brain regions, hindering discovery of broader patterns. Moreover, the findings may lack generalizability, as most studies rely on convenience samples with demographic imbalances^55,57^. Demographic and clinical factors like age, sex, and disease duration influence brain structure, raising the possibility that observed group differences reflect these confounds rather than true disease effects^26,58^. Since our deconfounding strategies appear insufficient, it is crucial to first establish that groups are more comparable in terms of their population background variation to ensure valid comparisons^25^. To address these limitations, we implemented a rigorous matching framework using propensity scores to balance demographic and clinical characteristics between subgroups^59^. In addition, we accompanied the careful participant matching by a pattern-matching approach to quantify differences between subgroups. Comparing the expression of well-established patterns of PD-related atrophy in smaller subgroups maximized our statistical power to detect widespread neural changes. This strategy opened up a more holistic view onto disease-related changes and allowed for the detection of complex structural patterns that might be missed when focusing on individual regions. These innovations provide a recipe for future studies to more accurately capture and interpret structural brain changes in complex brain disorders.

Alongside the strengths of our approach, some limitations inherent to characteristics of the PPMI data and study design should be acknowledged. Demographic imbalances required a disciplined matching protocol, which resulted in automatically determined subgroups for comparison, particularly in our targeted subgroup analyses. The geographic characteristics and ethnic composition of the PPMI cohort may also tie into the generalizability of our findings, as the sample overrepresents urban, highly educated, and predominantly white individuals with greater access to health care. Additionally, the cross-sectional design of our study limits the ability to draw conclusions about continuous disease progression or causality. Although we matched participants on key variables including disease duration, longitudinal follow-up will be essential to determine whether observed structural differences reflect truly biologically distinct neurodegenerative trajectories. While propensity score matching reduces key sources of bias, residual confounding from unmeasured variables such as medication use or comorbidities cannot be fully excluded. Comparable datasets with large sample sizes, deep phenotyping, harmonized imaging protocols, asyn SAA data, and several longitudinal brain-imaging sessions are currently lacking. As such resources become available, replication in more diverse and representative cohorts will be essential to validate and extend our current findings.

In conclusion, our pattern-matching strategy combined with careful modeling of individual heterogeneity revealed distinct MRI signatures among subgroups of PD patients. *LRRK2* pathogenic variants may be associated with a milder form of the PD phenotype. Lewy body presence is linked to a more aggressive neurodegeneration. These genetic-imaging findings support a view of PD as a spectrum of biologically distinct subtypes, each shaped by genetic and pathological factors. Given the relatively high prevalence of *LRRK2* pathogenic variants in some populations and their potential as therapeutic targets, our insights could facilitate the development of more precise prognostic models and personalized treatment strategies.

## Supplementary information

Supplementary Information

Reporting Summary

Supplementary Data 1

Supplementary Data 2

Supplementary Data 3

Supplementary Data 4

Supplementary Data 5

Transparent Peer Review file

## Data Availability

Data used in the preparation of this article were obtained in May 2023 from the Parkinson’s Progression Markers Initiative (PPMI) database (www.ppmi-info.org/access-data-specimens/download-data), RRID:SCR_006431. Researchers can request access to PPMI imaging, genetic, and clinical data by submitting a data use agreement at the PPMI website. For up-to-date information on the study, visit www.ppmi-info.org. All numerical data underlying the graphs and charts in the main figures are provided as individual Excel files in the Supplementary Data: source data for Figs. 1–5 are available in Supplementary Data 1–5, respectively. Code used for analysis is available as described in the Code Availability section.

## Code availability

The processing scripts and custom analysis software used in this work are available in a publicly accessible GitHub repository, along with examples of key visualizations in the paper: https://github.com/jakubkopal/LRRK2-MRI.
