# Supplementary material for: Carriers of LRRK2 pathogenic variants show a milder, anatomically distinct brain signature of Parkinson’s disease
Source: Commun Med (Lond). 2026 Jan 3;6:71. doi: 10.1038/s43856-025-01330-7 (PMC12868736; doi:10.1038/s43856-025-01330-7)
Supplement: Supplementary file 2 — Reporting Summary [file 43856_2025_1330_MOESM2_ESM.pdf]

nature portfolio

Corresponding author(s): Danilo Bzdok

Last updated by author(s): Oct 27, 2025

Reporting Summary

Nature Portfolio wishes to improve the reproducibility of the work that we publish. This form provides structure for consistency and transparency in reporting. For further information on Nature Portfolio policies, see our Editorial Policies and the Editorial Policy Checklist.

Statistics

For all statistical analyses, confirm that the following items are present in the figure legend, table legend, main text, or Methods section.

n/a	Confirmed	
☐	☒	The exact sample size (*n*) for each experimental group/condition, given as a discrete number and unit of measurement
☐	☒	A statement on whether measurements were taken from distinct samples or whether the same sample was measured repeatedly
☐	☒	The statistical test(s) used AND whether they are one- or two-sided<br>*Only common tests should be described solely by name; describe more complex techniques in the Methods section.*
☐	☒	A description of all covariates tested
☐	☒	A description of any assumptions or corrections, such as tests of normality and adjustment for multiple comparisons
☐	☒	A full description of the statistical parameters including central tendency (e.g. means) or other basic estimates (e.g. regression coefficient) AND variation (e.g. standard deviation) or associated estimates of uncertainty (e.g. confidence intervals)
☐	☒	For null hypothesis testing, the test statistic (e.g. *F*, *t*, *r*) with confidence intervals, effect sizes, degrees of freedom and *P* value noted<br>*Give P values as exact values whenever suitable.*
☒	☐	For Bayesian analysis, information on the choice of priors and Markov chain Monte Carlo settings
☒	☐	For hierarchical and complex designs, identification of the appropriate level for tests and full reporting of outcomes
☐	☒	Estimates of effect sizes (e.g. Cohen's *d*, Pearson's *r*), indicating how they were calculated

Our web collection on statistics for biologists contains articles on many of the points above.

Software and code

Policy information about availability of computer code

Data collection	Clinical, imaging, genetic, and biomarker data were collected as part of the multicenter Parkinson’s Progression Markers Initiative (PPMI) study. All participants underwent T1-weighted MRI acquired using standardized procedures and acquisition parameters across sites, and cerebrospinal fluid was collected for α-synuclein seed amplification assay (SAA). Data collection followed the PPMI study protocol (see www.ppmi-info.org).
Data analysis	The processing scripts and custom analysis software used in this work are available in a publicly accessible GitHub repository:<br>https://github.com/jakubkopal/LRRK2-MRI<br>The whole pipeline was written in Python (version 3.12.0), with the following external package:<br>scikit-learn (version: 1.3.1)<br>numpy (version: 1.26.4)<br>seaborn (version: 0.13.0)<br>scipy (1.13.1)<br>pandas (version: 2.1.1)<br>matplotlib (version: 3.8.0)<br>nilearn (version: 0.10.2)<br>Python (version: v3.8.12)<br>statsmodels (0.14.0)<br>BrainStat (0.4.2)

For manuscripts utilizing custom algorithms or software that are central to the research but not yet described in published literature, software must be made available to editors and reviewers. We strongly encourage code deposition in a community repository (e.g. GitHub). See the Nature Portfolio guidelines for submitting code & software for further information.

Data

Policy information about availability of data

All manuscripts must include a data availability statement. This statement should provide the following information, where applicable:

- Accession codes, unique identifiers, or web links for publicly available datasets
- A description of any restrictions on data availability
- For clinical datasets or third party data, please ensure that the statement adheres to our policy

Provide your data availability statement here.

Research involving human participants, their data, or biological material

Policy information about studies with human participants or human data. See also policy information about sex, gender (identity/presentation), and sexual orientation and race, ethnicity and racism.

Reporting on sex and gender	Sex (male/female) was recorded in PPMI based on clinical records at enrollment. Sex was included as a covariate in all statistical models because of known sex differences in Parkinson's disease risk and neurodegeneration. We did not collect or analyze gender identity, as these data are not available in PPMI. Sex-specific model parameters are provided in the Supplementary Materials. No sex-stratified subgroup analyses were performed due to limited sample sizes in some subgroups.
Reporting on race, ethnicity, or other socially relevant groupings	Race and ethnicity data are not available through the PPMI dataset used in this study; therefore, these variables could not be included in the analyses. As noted in the Discussion, the PPMI cohort overrepresents urban, highly educated, and predominantly white individuals with greater access to healthcare, which may limit generalizability of the findings. Demographic characteristics that were available (age, sex, disease duration, and site) are reported in Tables 1 and 2 and were used for propensity score matching to minimize population-related confounding.
Population characteristics	Participants were recruited as part of the Parkinson's Progression Markers Initiative (PPMI), a multicenter observational study. The study population included adults with sporadic Parkinson's disease (sPD), Parkinson's disease with LRRK2 pathogenic variants, non-manifesting LRRK2 variant carriers, and healthy controls without neurological disorders. Inclusion and exclusion criteria for all participant groups follow the PPMI protocol. Key demographic and clinical characteristics, including age, sex, and disease duration, are reported in Tables 1 and 2. There were demographic differences between subgroups (e.g., age, sex distribution, disease duration), which were addressed using propensity score matching to improve group comparability.
Recruitment	Participants were recruited through the PPMI, a multicenter observational study. Individuals with sporadic PD were enrolled within 2 years of diagnosis and initially untreated with dopaminergic medication; those with known pathogenic mutations were excluded. LRRK2 PD participants carried a pathogenic LRRK2 variant and were enrolled within 7 years of diagnosis. LRRK2 non-manifesting carriers were first-degree relatives of PD patients and had no clinical PD diagnosis. Healthy controls had no neurological disorders and no PD-related pathogenic variants. All PD diagnoses were confirmed by abnormal dopamine transporter imaging. Participants were included in this analysis only if MRI and relevant clinical data were available.
Ethics oversight	All data were obtained from the PPMI, which received ethics approval from institutional review boards at each participating site, and written informed consent was obtained from all participants at enrollment. This study involved secondary analysis of de-identified data and therefore required no additional ethics approval at our institutions. All research was conducted in accordance with the Declaration of Helsinki and relevant local regulations.

Note that full information on the approval of the study protocol must also be provided in the manuscript.

Field-specific reporting

Please select the one below that is the best fit for your research. If you are not sure, read the appropriate sections before making your selection.

☒ Life sciences ☐ Behavioural & social sciences ☐ Ecological, evolutionary & environmental sciences

For a reference copy of the document with all sections, see nature.com/documents/nr-reporting-summary-flat.pdf

Life sciences study design

All studies must disclose on these points even when the disclosure is negative.

Sample size	This study included 603 participants with available MRI and clinical data from the PPMI cohort: 293 sporadic PD, 77 LRRK2 PD, 94 LRRK2 non-manifesting carriers, and 139 healthy controls. No formal sample size calculation was performed, as all eligible participants with relevant data were included. To address demographic differences between subgroups and improve comparability, matched subsets were created using propensity score matching, resulting in smaller analytical samples for targeted subgroup analyses.
Data exclusions	Participants were included only if they had T1-weighted MRI that passed visual quality control and corresponding clinical and genetic data available through PPMI. Individuals with missing MRI, failed QC, or missing genotype or CSF SAA data were excluded from the relevant

analyses, as pre-specified in the study protocol. No post-hoc exclusions were made, and all exclusions were unrelated to the outcomes of interest (brain morphometry). Final sample sizes for each analysis are reported in Tables 1–2 and Figures 2–5.

Replication	This study is based on a single observational cohort; therefore, no experimental or technical replicates were applicable. Each participant represents one biological replicate. All analyses were performed using a standardized processing and modeling pipeline that was applied consistently across participant subgroups. The full code used for data processing, matching, and statistical analysis is publicly available to enable independent replication of the results (see Code availability). Propensity score matching algorithm was evaluated across range of caliper magnitudes as depicted in the Supplement.
Randomization	Randomization was not applicable, as this study involved secondary analysis of an observational cohort without experimental manipulation or allocation to intervention groups. Participants received standard clinical care as part of the PPMI study protocol.
Blinding	Blinding was not applicable, as this study analyzed previously collected observational data without treatment allocation or outcome assessment requiring concealment. MRI-based structural measures are objectively derived, and analysts had no influence on participant clinical status.

Reporting for specific materials, systems and methods

We require information from authors about some types of materials, experimental systems and methods used in many studies. Here, indicate whether each material, system or method listed is relevant to your study. If you are not sure if a list item applies to your research, read the appropriate section before selecting a response.

Materials & experimental systems

n/a	Involved in the study
☒	☐ Antibodies
☒	☐ Eukaryotic cell lines
☒	☐ Palaeontology and archaeology
☒	☐ Animals and other organisms
☒	☐ Clinical data
☒	☐ Dual use research of concern
☒	☐ Plants

Methods

n/a	Involved in the study
☒	☐ ChIP-seq
☒	☐ Flow cytometry
☐	☒ MRI-based neuroimaging

Plants

Seed stocks	*Report on the source of all seed stocks or other plant material used. If applicable, state the seed stock centre and catalogue number. If plant specimens were collected from the field, describe the collection location, date and sampling procedures.*
Novel plant genotypes	*Describe the methods by which all novel plant genotypes were produced. This includes those generated by transgenic approaches, gene editing, chemical/radiation-based mutagenesis and hybridization. For transgenic lines, describe the transformation method, the number of independent lines analyzed and the generation upon which experiments were performed. For gene-edited lines, describe the editor used, the endogenous sequence targeted for editing, the targeting guide RNA sequence (if applicable) and how the editor was applied.*
Authentication	*Describe any authentication procedures for each seed stock used or novel genotype generated. Describe any experiments used to assess the effect of a mutation and, where applicable, how potential secondary effects (e.g. second site T-DNA insertions, mosiacism, off-target gene editing) were examined.*

Magnetic resonance imaging

Experimental design

Design type	Structural MRI (T1-weighted) used to assess cortical thickness and subcortical volume
Design specifications	This study used high-resolution T1-weighted anatomical scans acquired during a single structural MRI session per participant in the PPMI cohort. No task, trial, or stimulus design was involved. Scans were collected across multiple sites using standardized acquisition parameters and were used to derive cortical thickness and subcortical volume measures for between-group comparisons.
Behavioral performance measures	No behavioral performance measures were collected during MRI acquisition, and no behavioral tasks were performed in the scanner. Clinical assessments (e.g., MDS-UPDRS III, MoCA) were acquired outside the MRI session and used only for secondary correlation analyses.

Acquisition

Imaging type(s)	Structural MRI (high-resolution 3D T1-weighted sequence; MP-RAGE/IR-FSPGR equivalent) acquired using harmonized acquisition protocols across PPMI study sites.
Field strength	3 T
Sequence & imaging parameters	High-resolution T1-weighted images were acquired sagittally with 1.0 mm isotropic voxels (slice thickness 1.0 mm; 256 × 256 matrix; 256 mm field of view; ~192 slices; anterior–posterior phase encoding). Acquisition parameters (including TR, TE, and flip angle) followed the standardized PPMI protocol and varied slightly by scanner vendor (MP-RAGE or IR-FSPGR equivalent). Typical scan duration was approximately 7 minutes.
Area of acquisition	Whole-brain structural MRI coverage, including cortex and subcortical structures.

Diffusion MRI ☐ Used ☒ Not used

Preprocessing

Preprocessing software	Structural MRI data were processed using FreeSurfer v7.2 to derive cortical thickness and subcortical volume measures.
Normalization	Cortical thickness and subcortical volume measures were computed in each participant's native anatomical space using FreeSurfer. No additional spatial normalization was performed prior to analysis. For statistical modeling, regional morphometric measures were standardized (z-scored) across the study sample.
Normalization template	FreeSurfer's standard surface-based registration to the fsaverage template was used to enable inter-subject alignment of cortical thickness maps. Subcortical segmentation was performed using probabilistic atlas priors in native space. No additional nonlinear spatial normalization was applied for morphometric analyses.
Noise and artifact removal	All MRI scans underwent visual quality control to identify and exclude images with major motion artifacts or segmentation failures. FreeSurfer's automated topology correction and surface refinement procedures were used to address minor surface reconstruction artifacts, and only scans passing final QC were included in downstream analyses.
Volume censoring	Not applicable. No temporal MRI sequences were acquired, and no volumes required omission due to motion or other artifacts beyond standard quality control exclusions.

Statistical modeling & inference

Model type and settings	Region-wise linear regression models were used to estimate associations between PD diagnosis (or subgroup status) and morphometric measures. Models included age, sex, scanning site, and where applicable disease duration or total intracranial volume as covariates. Statistical analyses were performed in Python (v3.10) using statsmodels (v0.14) and scikit-learn (v1.3.1).
Effect(s) tested	We tested group effects related to sporadic PD versus controls, LRRK2 PD versus sporadic PD, LRRK2 non-manifesting carriers versus controls, and α-synuclein SAA positive versus negative participants. Structural differences between predicted and observed morphometry were additionally evaluated using paired t-tests. Correlations with UPDRS-III, MoCA, and PD polygenic risk scores were assessed using Pearson's or partial correlation as appropriate.

Specify type of analysis: ☐ Whole brain ☒ ROI-based ☐ Both

Anatomical location(s)	68 cortical parcels according to the Desikan-Killiany atlas and 14 subcortical parcels according to Harvard-Oxford atlas
Statistic type for inference (See Eklund et al. 2016)	Our analysis was conducted using linear models predicting brain morphometry based on disease status, sex, age, scanner type, and disease duration.
Correction	FDR corrections were used for testing linear association strength when applicable.

Models & analysis

n/a	Involved in the study
☒	☐ Functional and/or effective connectivity
☒	☐ Graph analysis
☐	☒ Multivariate modeling or predictive analysis

Multivariate modeling and predictive analysis	This study includes predictive analysis by applying region-wise linear models trained on matched sporadic PD vs control samples to independent subgroups to estimate expected morphometry and quantify prediction deviations.
